# Supplementary material for: An Explorative Analysis of ABCG2/TOP-1 mRNA Expression as a Biomarker Test for FOLFIRI Treatment in Stage III Colon Cancer Patients: Results from Retrospective Analyses of the PETACC-3 Trial
Source: Cancers (Basel). 2020 Apr 15;12(4):977. doi: 10.3390/cancers12040977 (PMC7226226; doi:10.3390/cancers12040977)

Supplementary Materials

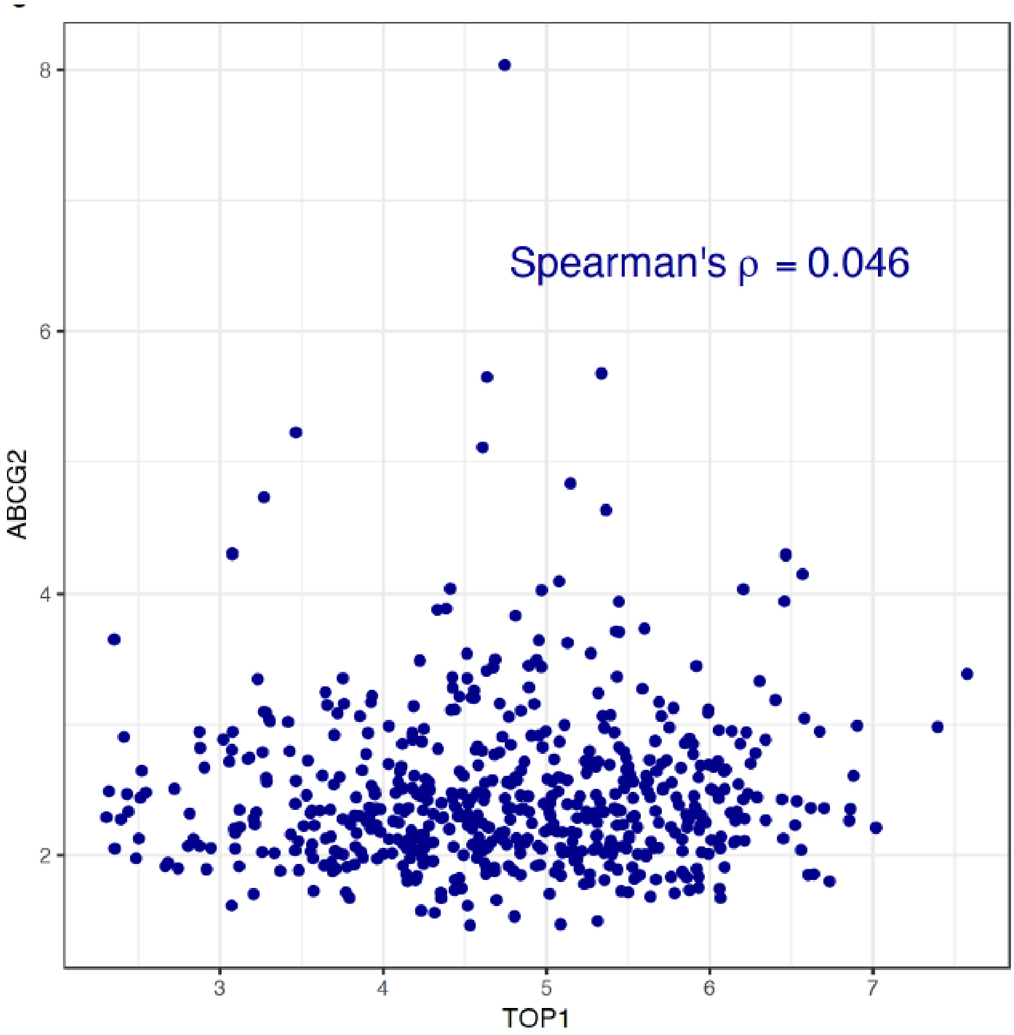

Figure S1. Scatter plot of log2-expression levels of ABCG2 and TOP1 genes.

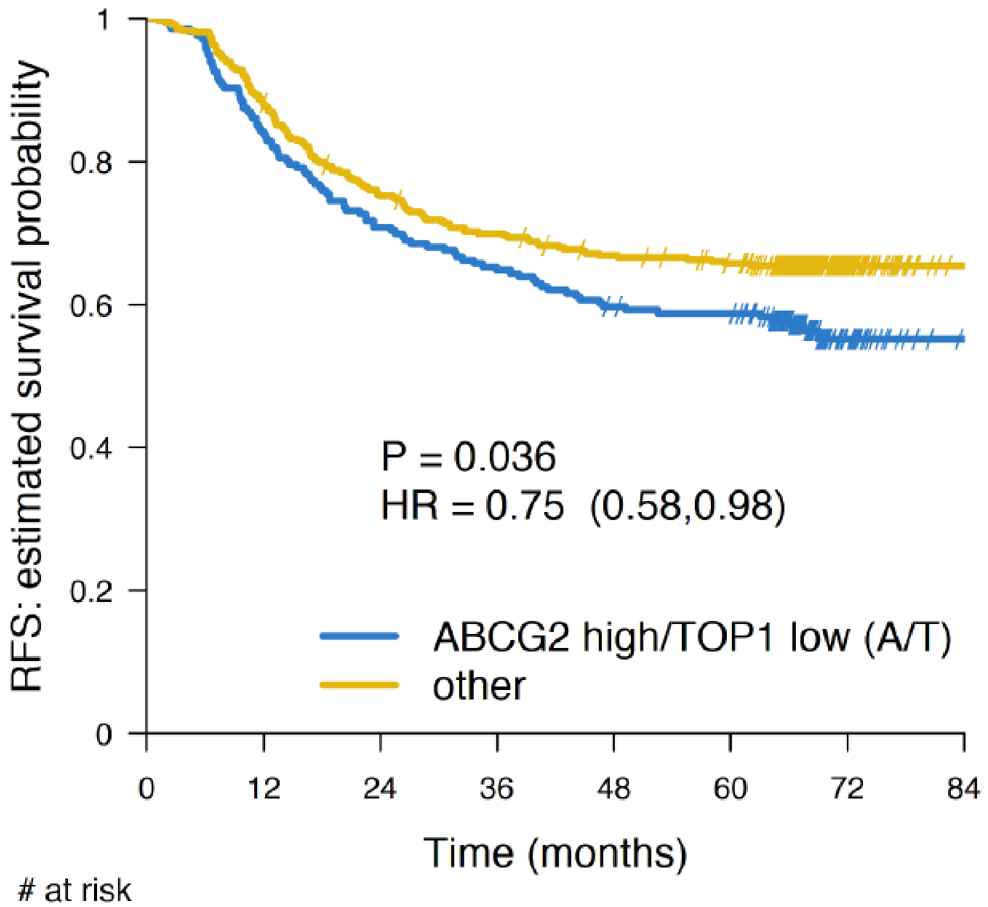

# at risk								
A/T	216	182	153	140	128	125	21	1
other	364	319	272	252	238	229	46	4

Figure S2. RFS stratified by biomarker in whole stage III. Survival plot (Kaplan-Meier estimates) of "resistant" and "sensitive" groups, as defined by the test, in the whole study population (all stage III, $n = 580$).

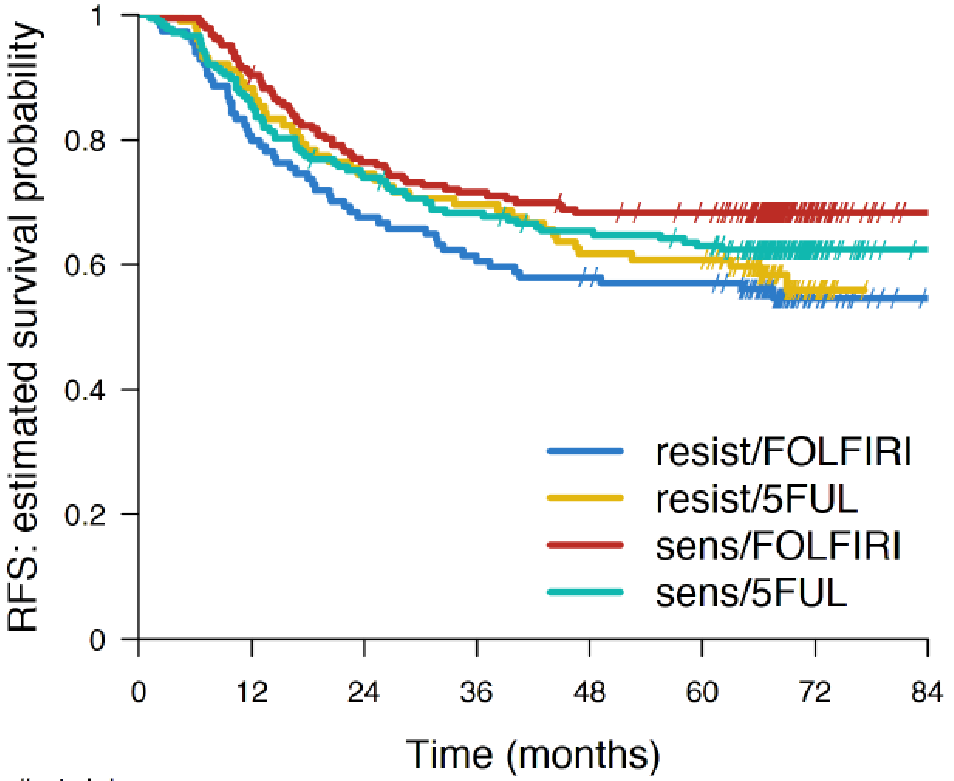

# at risk								
R/F.IRI	114	92	77	69	65	63	12	1
R/5FUL	102	90	76	71	63	62	9	0
S/F.IRI	187	168	142	133	126	122	22	1
S/5FUL	177	151	130	119	112	107	24	3

Figure S3. RFS stage III stratified by biomarker and treatment. Survival plot (Kaplan-Meier estimates) of "resistant" and "sensitive" groups under FOLFIRI treatment, and all 5FUL-only treated patients were pooled. While the "sensitive" patients treated with FOLFIRI seem to fare best among the three groups, the difference is not statistically significant when compared with 5FUL-treated group (HR: 0.77, 95%CI: (0.56–1.06); p = 0.11). Abbreviations: "resistant" under FOLFIRI = "resist/FOLFIRI" = "R/F.IRI", "sensitive" under 5FUL = "sens/5FUL" = "S/5FUL" etc.

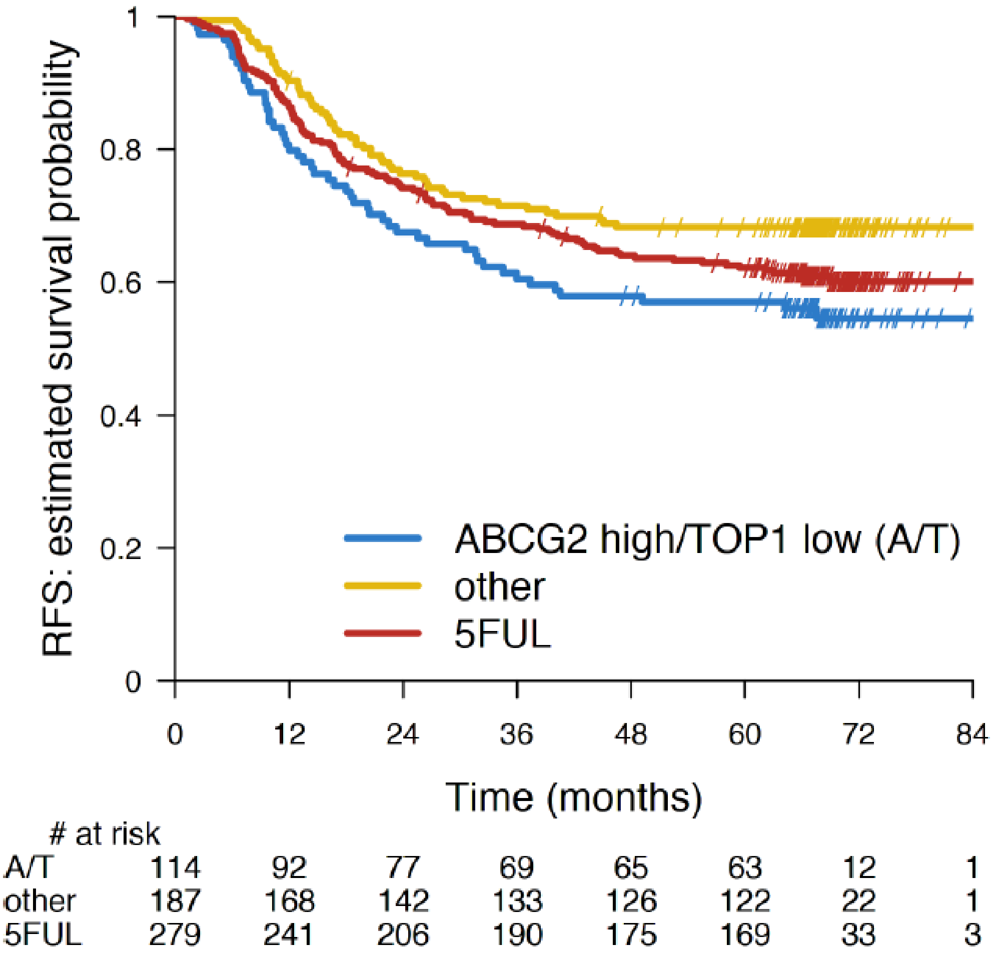

Figure S4. RFS in FOLFIRI by biomarker vs whole 5FUL arm. In both treatment arms, the patients were dichotomized by the biomarker test. However, although not reaching statistical significance, the "sensitive" patients in the FOLFIRI group appeared to have a better RFS than any of the other groups.

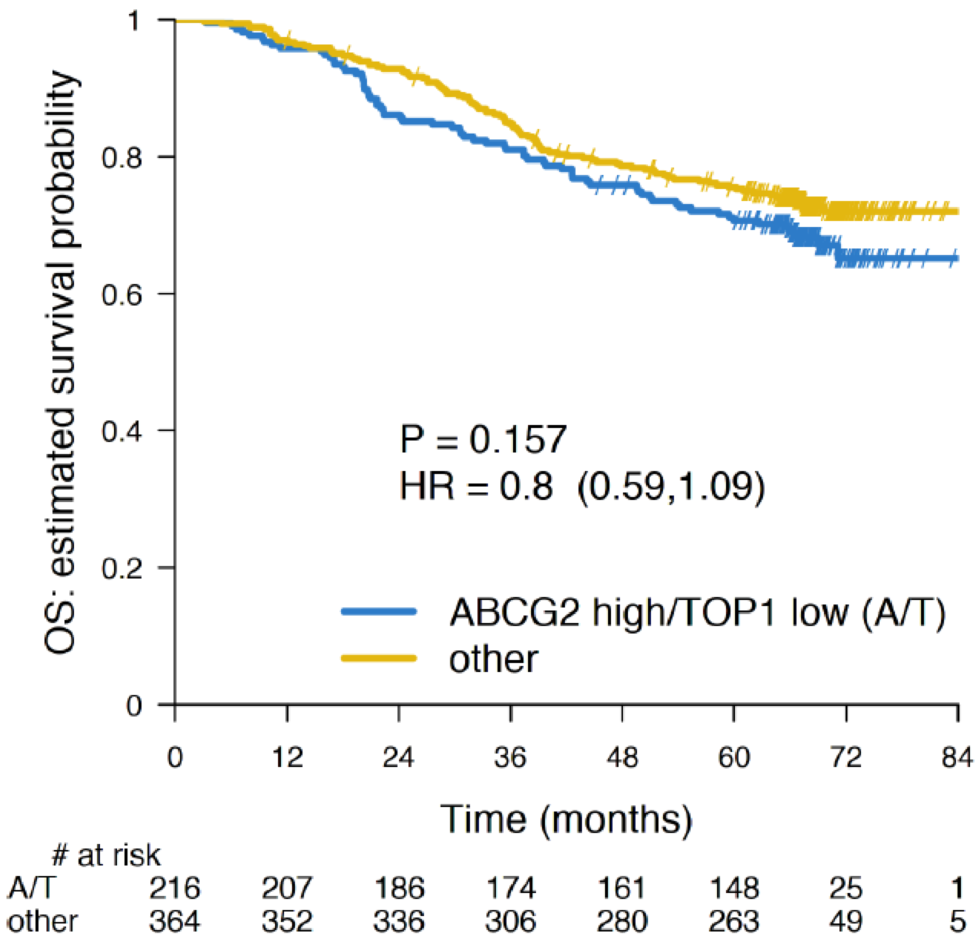

Figure S5. OS by biomarker in whole Stage III. OS of all 580 CC patients dichotomized by the biomarker.

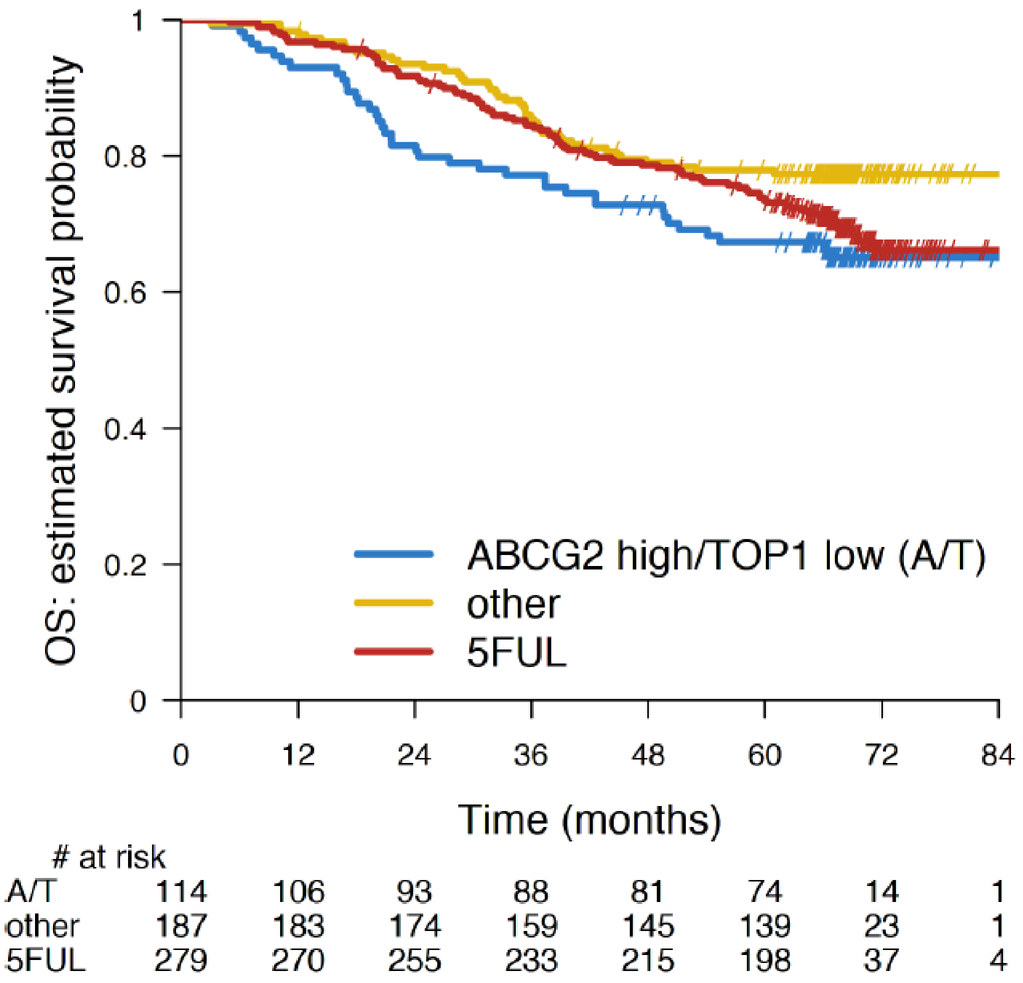

Figure S6. OS by biomarker in FOLFIRI arm vs 5FUL arm. This Figure shows the associations between OS and the three subgroups (all 5FUL, FOLFIRI “sensitive” and FOLFIRI “resistant”, respectively. No significant differences were found between the three groups.

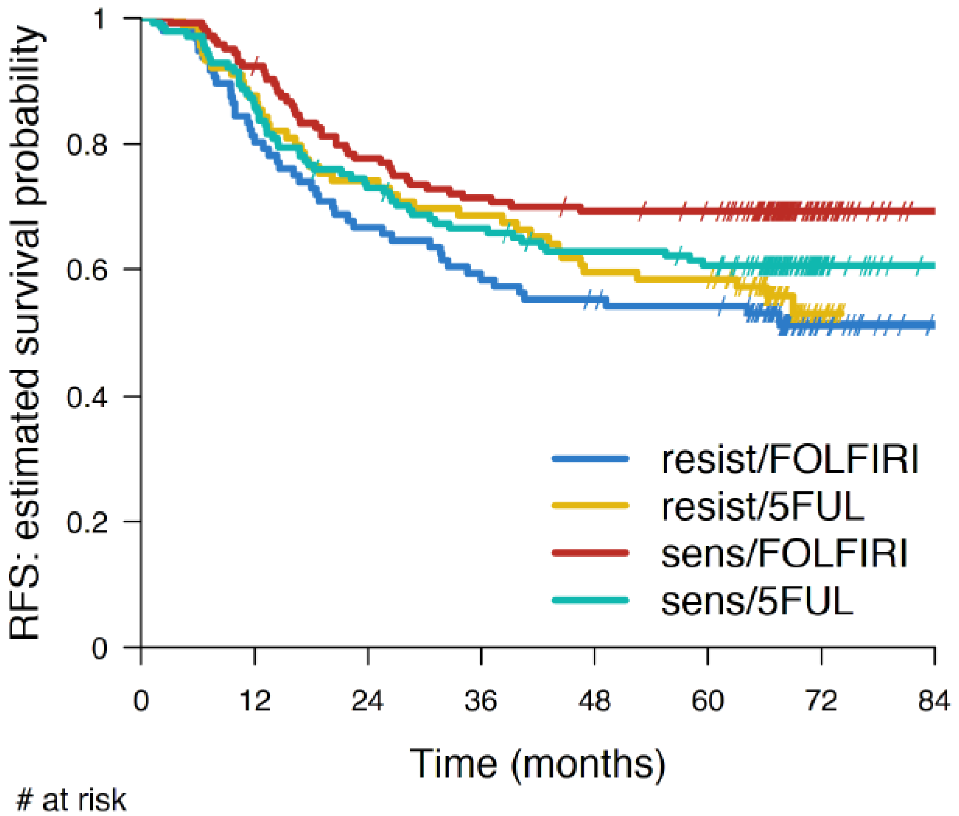

# at risk								
R/F.IRI	96	78	64	56	52	50	11	1
R/5FUL	89	78	66	61	53	52	7	0
S/F.IRI	144	132	111	102	98	95	20	1
S/5FUL	141	121	102	92	85	81	17	3

Figure S7: RFS stage III/MSS stratified by biomarker and treatment. RFS in MSS patients. The patients were divided into 4 groups representing "sensitive" and "resistant" patients in each of the two treatment arms. Abbreviations: "resistant" under FOLFIRI = "resist/FOLFIRI" = "R/F.IRI", "sensitive" under 5FUL = "sens/5FUL" = "S/5FUL" etc.

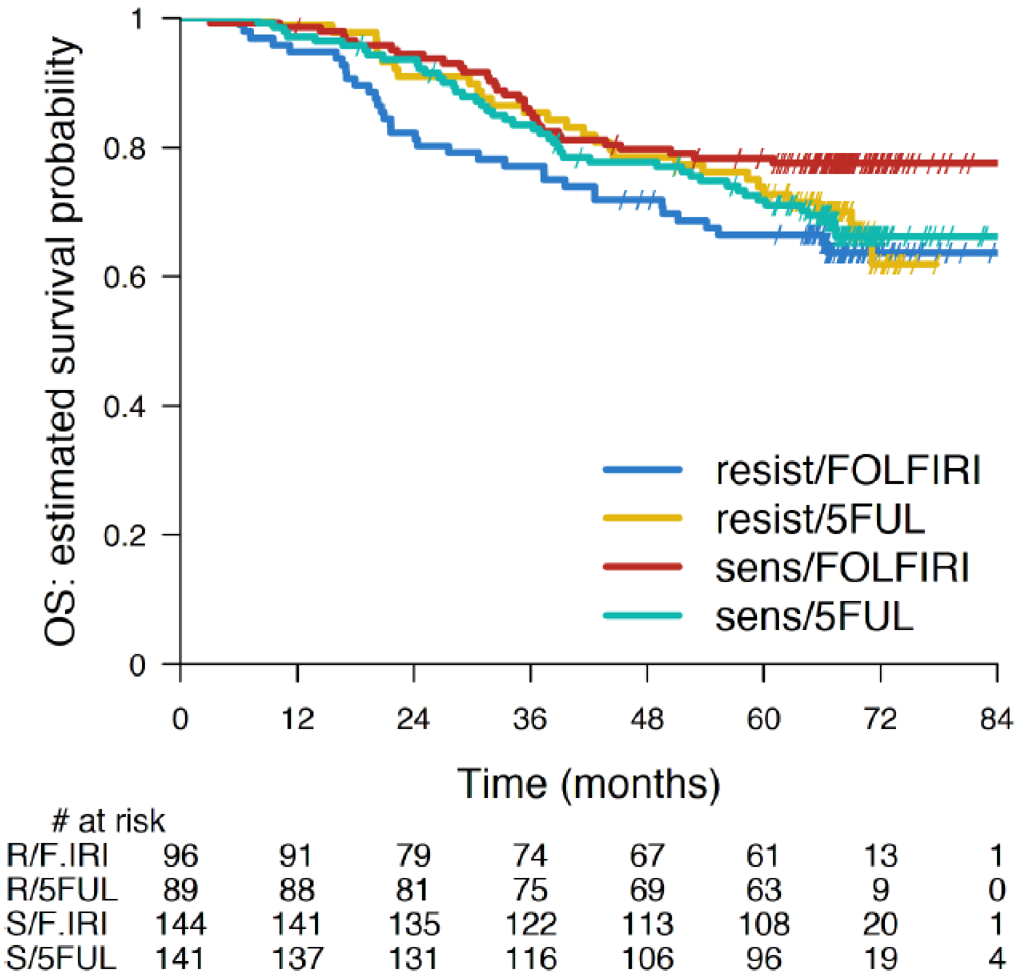

Figure S8. OS for stage III/MSS stratified by biomarker and treatment. OS in MSS patients. The patients were divided into 4 groups representing "sensitive" and "resistant" patients in each of the two treatment arms.

Multivariable Cox regression analyses

The following sections present the results of multivariable analyses and the visualization of the estimated coefficient profiles (from `glmnet` R package). To understand the coding of the variables, the following definitions are needed:

- atb2: ABCG2/TOP1 biomarker, a binary factor with "resistant" and "other" levels
- MSI: MSI-H status, a binary factor with "MSI-H" and "MSS" levels
- BRAF: BRAF V600E mutation status, a binary factor with "mut" and "wt" levels for mutant and wild type, respectively
- KRAS: KRAS codon 12 and 13 mutation status, a binary factor with "mut" and "wt" levels for mutant and wild type, respectively
- site: tumor site, a binary factor with "left" and "right" levels
- mucinous: mucinous histology, a binary factor with "yes" and "no" levels

For each model, full output is provided.

2.1. In all Stage II

```
Call:
coxph(formula = Surv(rfs.time, rfs.event) ~ atb2 + MSI + BRAF +
    KRAS + site, data = C)

             coef exp(coef) se(coef)     z     p
atb2other -0.284     0.753    0.146 -1.94 0.052
MSIMSS     0.518     1.679    0.293  1.77 0.077
BRAFwt    -0.617     0.540    0.278 -2.22 0.026
KRASwt    -0.342     0.710    0.151 -2.27 0.023
siteright  0.125     1.133    0.155  0.81 0.419

Likelihood ratio test=17.1  on 5 df, p=0.00435
n= 515, number of events= 196
   (65 observations deleted due to missingness)
```

Coefficients profiles from LASSO penalized regression:

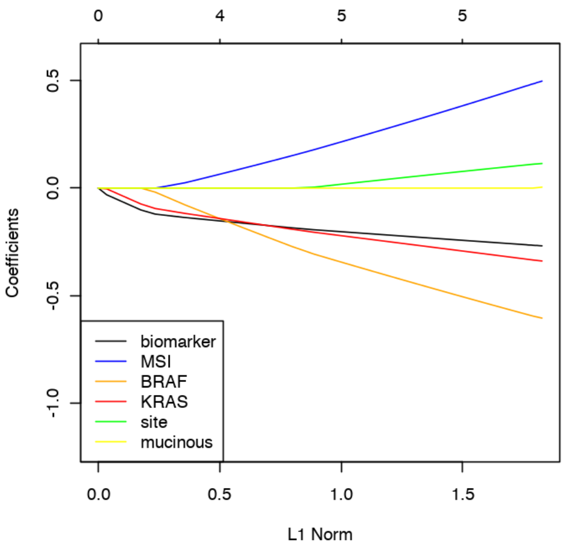

2.2. In Stage III/MSS

```
Call:
coxph(formula = Surv(rfs.time, rfs.event) ~ atb2 + BRAF + KRAS +
    site + mucinous, data = C[i.mss, ])

               coef exp(coef) se(coef)     z     p
atb2other   -0.3335    0.7164   0.1516 -2.20 0.028
BRAFwt      -0.5920    0.5532   0.3198 -1.85 0.064
KRASwt      -0.3938    0.6745   0.1559 -2.53 0.012
siteright    0.0753    1.0782   0.1627  0.46 0.643
mucinousyes  0.1238    1.1317   0.1998  0.62 0.536

Likelihood ratio test=16.5  on 5 df, p=0.0056
n= 462, number of events= 181
   (8 observations deleted due to missingness)
```

Coefficients profiles from LASSO penalized regression:

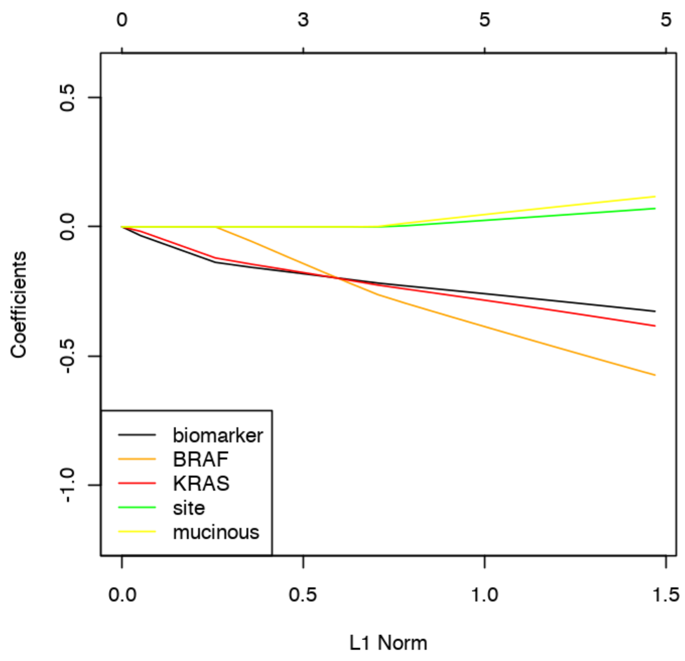

2.3. In Stage III/FOLFIRI

With selected variables:

```
Call:
coxph(formula = Surv(rfs.time, rfs.event) ~ atb2 + MSI + BRAF +
    KRAS + site, data = C[i.folfiri, ])

            coef exp(coef) se(coef)     z     p
atb2other -0.476     0.621    0.206 -2.32 0.020
MSIMSS     0.505     1.657    0.398  1.27 0.205
BRAFwt    -0.940     0.391    0.374 -2.51 0.012
KRASwt    -0.437     0.646    0.216 -2.03 0.043
siteright  0.263     1.301    0.213  1.24 0.216

Likelihood ratio test=17.6  on 5 df, p=0.00343
n= 262, number of events= 97
   (39 observations deleted due to missingness)
```

Coefficients profiles from LASSO penalized regression:

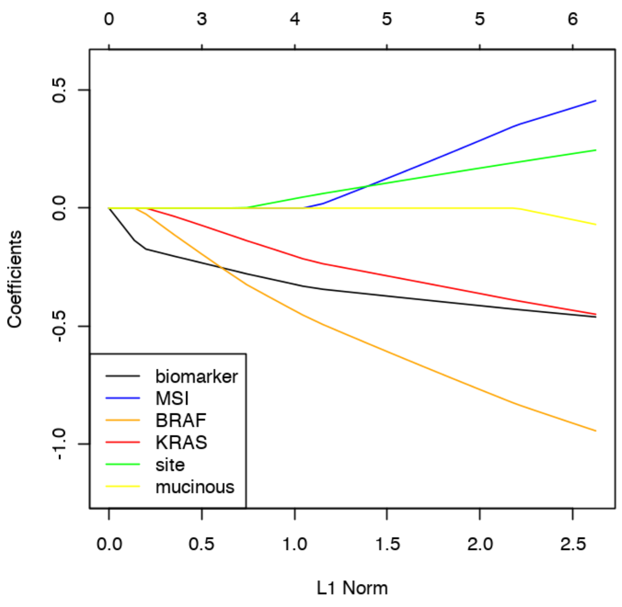

2.4. In Stage III/MSS/FOLFIRI

```
Call:
coxph(formula = Surv(rfs.time, rfs.event) ~ atb2 + BRAF + KRAS +
    site, data = C[intersect(i.mss, i.folfiri), ])

            coef exp(coef) se(coef)     z      p
atb2other -0.557     0.573    0.215 -2.60 0.0094
BRAFwt    -1.014     0.363    0.416 -2.44 0.0147
KRASwt    -0.461     0.631    0.222 -2.07 0.0380
siteright  0.233     1.263    0.218  1.07 0.2847

Likelihood ratio test=18.1  on 4 df, p=0.00118
n= 236, number of events= 89
   (4 observations deleted due to missingness)
```

Coefficients profiles from LASSO penalized regression:

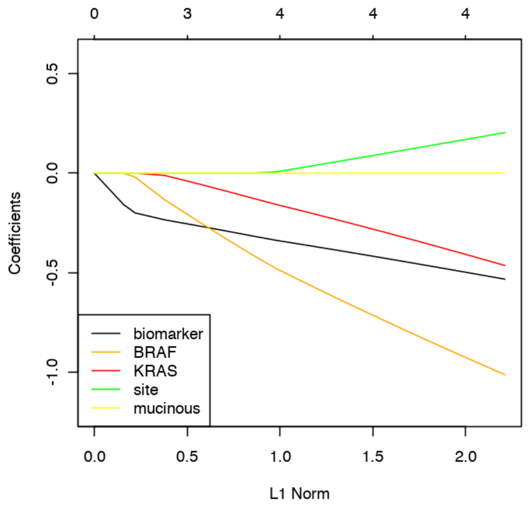

Supplement: Supplementary file 1 [file cancers-12-00977-s001.pdf]
